# The Hypothalamic-Pituitary-Thyroid Axis in Infants and Children: Protection from Radioiodines

**DOI:** 10.1155/2014/710178

**Published:** 2014-05-25

**Authors:** Jeffrey Fisher, Xiaoxia Yang, Curtis Harris, Igor Koturbash, Annie Lumen

**Affiliations:** ^1^National Center for Toxicological Research, US FDA, 3900 NCTR Road, Jefferson, AR 72079, USA; ^2^College of Public Health, University of Georgia, 115 DW Brooks Dr, Barrow Hall, Room 001B, Athens, GA 30602, USA; ^3^Department of Environmental and Occupational Health, Fay W. Boozman College of Public Health, University of Arkansas for Medical Sciences, 4301 West Markham Street, Slot 820-11, Little Rock, AR 72205, USA

## Abstract

Potassium iodide (KI) is recommended as an emergency treatment for exposure to radioiodines, most commonly associated with nuclear detonation or mishaps at nuclear power plants. Protecting the thyroid gland of infants and children remains a priority because of increased incidence of thyroid cancer in the young exposed to radioiodines (such as ^131^I and ^133^I). There is a lack of clinical studies for KI and radioiodines in children or infants to draw definitive conclusions about the effectiveness and safety of KI administration in the young. In this paper, we compare functional aspects of the hypothalamic-pituitary-thyroid (HPT) axis in the young and adults and review the limited studies of KI in children. The HPT axis in the infant and child is hyperactive and therefore will respond less effectively to KI treatment compared to adults. Research on the safety and efficacy of KI in infants and children is needed.

## 1. Introduction

Several governmental and scientific bodies are dedicated to ensuring protection of the public from radiation. The International Commission on Radiological Protection was created in 1928 by the International Congress of Radiology to advance the science of radiation protection for the public by publishing peer reviewed radiation articles. The United States Atomic Regulatory Commission was established in 1946 with a mission to encourage nuclear power and also protect the public. Eventually strong concerns about the dual roles of the Commission resulted in Congress dissolving this Commission and forming another, the United States Nuclear Regulatory Commission (USNRC). The USNRC began operation in 1975 and is responsible for protection and measurement, with regulatory involvement in nuclear facilities and protection of public health and safety. In 1955, the General Assembly of the United Nations established the United Nations Scientific Committee on the Effects of Atomic Radiation (UNSCEAR), which releases reports on important current topics of concern such as the Fukushima-Daiichi nuclear power plant accident. In 1964, the US congress chartered the National Council on Radiation Protection and Measurements with a mission to develop and disseminate guidance and recommendations on radiation protection and measurement. This group is well known for producing technical documents concerning a vast array of radiation topics.

Radioisotopes of iodines (stable elemental iodine (^127^I) and radioactive forms of iodine (such as ^131^I) are converted in the body to stable iodide (^127^I^−^) and radioiodide (^131^I^−^), primarily in the gut. Iodine in the diet refers to several inorganic forms (e.g., iodates) that are converted in the body to iodide) are produced in large quantities by fission reactions of uranium atoms in nuclear reactors and either plutonium or uranium atoms in nuclear detonations. The radioisotopes of iodines are subsequently released into the environment. At Three Mile Island, 6 × 10^11^ Bq was released into the atmosphere over several hours, while at Chernobyl 10 × 10^17^ Bq was released over a 10-day period, and most recently at Fukushima, several releases occurred totaling 10 × 10^16^ Bq [33]. Once in the environment, the radioactive iodines can be inhaled or ingested by consumption of contaminated food and milk. The threat of nuclear detonations or nuclear industrial accidents resulted in laboratory experiments to characterize the health hazards of radionuclides throughout the late 1940s into the 1960s [34–37]. Fresh cow's milk was considered to be a primary source of radionuclides after cows forage on contaminated vegetation. The first radioiodine and stable iodine (^125^I) experiments with cows to simulate a nuclear detonation or nuclear industrial accident were conducted by Bustad et al. [34]. The authors demonstrated that increasing the intake of stable or dietary iodine resulted in less radioiodide dose in the thyroid gland and milk. The use of stable iodine as a blocking agent for thyroidal uptake of radioiodide was an area of active research in the 1950s and 1960s [16, 30, 38]. Unknown at this time, iodide in systemic circulation, radioactive or stable, is sequestered into the thyroid gland and mammary tissue by the sodium iodide symporter protein (NIS), a basolateral transport protein. The stable form of systemic iodide competes with radioiodide for transport into the thyroid gland via the NIS. Additionally excess stable systemic iodide can cause a transient shut-down in the thyroid gland referred to as the Wolff-Chaikoff effect [39]. The mechanism of iodide action may involve posttranslational modification of the NIS [40]. Once in the thyroid gland excess stable iodide can also bind to thyroglobulin, the protein backbone for synthesis of thyroid hormones, and reduce the extent of binding of radioiodides to thyroglobulin.

Thyroid cancer in children and adolescents is one of the most severe health outcomes resulting from releases of radioiodines after a nuclear mishap or detonation [33]. Irradiation of the thyroid gland may also result in nodules or hypothyroidism [41]. Children's increased sensitivity to thyroidal radiation is one of the most important scientific findings from the Chernobyl nuclear accident. The rates of thyroid cancer were greater than expected in children exposed to radioactive isotopes of iodide [42, 43]. A workshop report [44] found evidence for mild goiter in the regions' children suggesting mild to moderate iodide deficiency. Some of the conclusions in the workshop report were that iodide deficient children with goiter may result in less radiation dose per mg weight of the thyroid gland or an altered distribution of radioiodides within the thyroid gland may occur. The report also noted that thyroidal uptake of systemic radioiodide may be greater in iodide deficient children compared to euthyroid children. In addition to radioiodide dosimetry concerns in iodide deficient children the thyroid glands of these children may be more susceptible to exposure of radioiodines. Potassium iodide (KI), a thyroidal radioiodide blocking agent, is recommended by the United States Food and Drug Administration (US FDA) as a drug to reduce the thyroid radioiodide dose in children and adults [45].

In this paper, we integrate interdisciplinary information from health physics, basic and clinical thyroid endocrinology, physiology, public health, and mathematical modeling. This review focuses on the biology of the hypothalamic-pituitary-thyroid (HPT) axis in children, selected clinical and field studies with KI and radioiodine tracer, and current knowledge about KI use in children. Data gaps are identified and research is recommended.

## 2. Aspects of the HPT Axis in Adults and Children

Protecting children from the potential harmful effects of radioiodines, drugs, or chemicals is not as simple as treating children as “small adults.” How children differ from adults in their overall kinetic and dynamic responses to drugs and chemicals remains a concern for conducting appropriate safety or risk assessments [46]. Growth from birth to adolescence alters, to varying degrees, the pharmacokinetics of therapeutic drugs and chemicals [46]. Several physiological processes undergo maturation including breathing rate, cardiac output, blood flows to tissues and organs, and organ or tissue volumes.  Table 1 provides examples of some important physiological changes that occur during maturation of infants and children. On a per Kg basis, cardiac output, ventilation rates, blood flow rates to organs, and organ volume are greater in early life (Table 1). Other aspects of physiology gradually increase with age such as glomerular filtration (Table 1) and kidney transporter proteins [47], which both affect the pharmacokinetics of drugs and chemicals. Enzyme maturation can be complex. For example, Cytochrome P450 (CYP) CYP3A is an important drug metabolizing enzyme. One isoform (CYP3A7) is active* in utero* and decreases the first week of life, while another isoform, CYP3A4, increases rapidly the first year of life [48]. Much is known about age-dependent changes in gross physiology; however, no data were found on the blood flow rate to the thyroid gland in infants and children. Measurements of blood flow rate for the thyroid gland in infants and children would help clarify if the rate of delivery of systemic radioiodide to the thyroid gland (normalized to the weight of the thyroid gland) is different than in adults.

Iodine is an essential element for life and its retention in the body is controlled by urinary excretion and sequestration into the thyroid gland by the NIS protein. Iodide can be sequestered into other NIS containing tissues (e.g., mammary tissue and gut lumen), but organification does not take place. Transfer of iodide (and to some degree thyroid hormones) to the nursing infant represents an important physiological process for dietary iodide intake for the nursing infant [49]. In the body, systemic levels of iodide originate from dietary ingestion of iodide and the release of iodide atoms from deiodination of thyroid hormones. The majority of iodide is excreted in urine with a minor loss to feces [50].

The function of the HPT axis has been studied by independently evaluating several aspects of the HPT axis. This includes measuring the uptake of trace amounts of radioiodide into the thyroid gland, intake of stable iodide, the excretion of stable iodide into urine, serum levels of stable iodide, thyroid pools of stable iodide, and rates of thyroid hormone secretion. The state of knowledge about the age-dependent changes in the HPT axis is discussed below.

### 2.1. Radioactive Iodide Uptake (RAIU) (Table 2)

Tracking trace amounts of radioactive iodide uptake (RAIU) into the thyroid gland in a clinical setting is expressed as percent of administered radioiodine dose. This measurement reflects the ability of the thyroid gland to sequester radioiodide and is a fundamental functional aspect of the thyroid gland. The measured fraction of administered radioiodine dose in the thyroid gland is a consequence of NIS mediated transport of radioiodide and its organification.

In a study conducted in the United States, Fields et al. reported a mean 24 hr RAIU value of 31.1% for 60 children [9], which did not differ from the mean 24 hr RAIU value for 64 euthyroid adults (32.2%). The same authors reported a mean 24 hr RAIU value of 76.1% for hyperthyroid adult (*n* = 62), which compares favorably to their findings for hyperthyroid children (87%, *n* = 5). Fields et al. stated that for euthyroid children under the age of 4 increased “hyperactivity” of the thyroid gland was evident and this “hyperactivity” may persist until age 9 [9]. These authors also stated that thyroid activity in older children aged 10 to 18 was similar to adults. Fields et al. also summarized 8 child RAIU studies reported from the late 1940s to early 1950s [9]. In general these earlier studies reported RAIU values less than the values Fields et al. reported [9], except for premature infants and newborns. Van Middleworth reported a mean RAIU value of 69.7% in 7 newborns from the United States [10] and Martmer reported a mean RAIU value of 38.6% for 16 premature infants from the United States [11].

A few RAIU studies have been reported on children since the 1950s. Twenty-four-hour RAIU values for 5 children from the United States, 2 years of age, were reported to range from 15 to 23% [12]. Twenty-six children, aged 9 to 15 years, had a mean 24 hr RAIU value of 52.9% [13]. Ingenbleek and Beckers reported a mean 24 hr RAIU value of 39.6% for children aged 1.5 to 2 years (*n* = 6) from the Republic of Senegal [14]. Cuddihy reported a mean RAIU value of 23.5% in 4 children aged 7 to 9 years from the United States [15].

Sternthal et al. determined a mean 24 hr RAIU value of 20.0% in adults from the United States [16]. Malvaux et al. reported a mean 24 hr RAIU value of 41.7% for Belgium adults [13]. Greer et al. reported a range of RAIU values in 37 adults from the United States of 18.1 to 21.6% [18]. In a recent German study, a mean RAIU value of 24.8% is reported for 27 adults [17].

A wide range of RAIU values are reported for children and adults. RAIU values reflect the ability of the thyroid gland to form precursor thyroid hormones (organify radioiodide) and the functionality of the NIS symporter proteins. Some studies show that the RAIU in the young is greater than in adults (Table 2) suggesting that infants and children may be at a greater risk of radiation exposure. An important confounding factor for interpreting these studies is the dietary iodine status. RAIU is well known to increase with decreasing dietary iodine.

### 2.2. Thyroidal Clearance of Radioactive Iodide (Table 2)

Another measure of thyroidal uptake of radioiodide is the thyroidal clearance of radioiodide, defined as a volume of serum or plasma cleared of radioiodide per unit time and is calculated by monitoring radioiodide uptake into the thyroid gland and clearance of radioiodide from the serum. Malvaux et al. reported thyroidal radioactive iodide clearance values of 30.3 and 25.3 mL/min for adolescents and adults [13] and when expressed as mL/min/kg, the values were 0.80 and 0.35. The authors also calculated 24 hr accumulation rates of dietary (stable) iodide in the thyroid gland of 1.0 and 0.65 *μ*g/kg/day for adolescents and adults. Ponchon et al. investigated the iodide kinetics in 21 newborns and infants [19], and pharmacokinetic parameters were compared to values reported earlier for adolescents and adults [13]. The thyroidal clearance of iodide, expressed in units of mL/min/kg, was found to decrease with increasing age. Ingenbleek and Beckers reported thyroidal radioiodide clearance values of 21.5 mL/min for normal children and 7.3 mL/min for malnourished children [14].

Thyroidal clearance of radioactive iodide (uptake of radioiodide into the thyroid gland and its clearance from blood) appears to be a better measure of the thyroidal uptake of radioiodide than RAIU because the rate at which this happens is captured and can be used in quantitative and dynamic evaluations of radioiodide dosimetry. These studies indicate that the uptake of thyroidal iodide occurs at a faster rate in the young than adults when normalized to body weight.

### 2.3. Urinary Excretion of Iodide (Table 2)

The neonatal kidneys are anatomically and functionally immature, exhibiting a disproportionately low glomerular filtration rate (about 30% of the adult level) and relatively low blood flow compared to the adult [51, 52]. The level of glomerular filtration increases very rapidly, especially within the first three months of life [52].

Age related differences in renal clearance of iodide from the plasma or serum have been documented. Oddie et al. evaluated dietary iodide in many subjects from Australia and the United States, using a linear statistical model [53]. Despite the immature glomerular filtration, the authors concluded that the renal clearance of iodide decreased with increasing age by about 0.7% per year. Also, hypothyroid individuals had a lower renal clearance rate of iodide compared to euthyroid individuals. Malvaux et al. reported renal clearance values of 25.5 and 29.7 mL/min for adolescents and adults and on a mL/min/kg basis, the values were 0.65 and 0.41 for adolescents and adults [13].

Similar to the thyroidal clearance of iodide, the renal clearance of iodide was also found to be higher in the young, particularly between the age of 3 weeks to 6 months [19]. When normalized for body weight, the young excrete iodide in urine at a greater rate than adults, which is inconsistent with maturation of glomerular filtration. These observations suggest that other biological determinants, perhaps age-dependent, are important for kidney function such as protein transporters.

### 2.4. Iodine Intake

Adequate intake levels for iodine estimated for the first year of life (110 to 130 *μ*g/day, [54]) are not much different than the recommended dietary allowances for adults (150 *μ*g/day, [54]) suggesting that on a body weight basis the HPT axis for the young is “accelerated.” In the United States, the pediatric intake of iodine, on a *μ*g/kg/day basis, is greater than adults [55]. In children of 6 years of age or under, the lower and upper bound for iodine intake ranged from 144 to 280 *μ*g/person/day and in adults 40 to 65 years of age, 138 to 284 *μ*g/person/day.

There is a dearth of data on direct measurements of iodide in serum or whole blood. A recent study in the United States [56] using modern analytical methods reported that median serum iodide concentrations were 8 *μ*g/L for fetal cord blood and 2.58 *μ*g/L for maternal blood serum. In China the reverse was reported, using modern analytical methods and whole blood samples. The mean infant iodide blood concentration was 15.7 *μ*g/L (range of 12.5 to 21.7 *μ*g/L) compared to adult mean serum iodide concentration of 110 *μ*g/L (range of 14.1 to 812 *μ*g/L) [57]. During the first year of life for infants in Belgium, the serum iodide levels were 2.2 to 6 *μ*g/L and for adults, near 1 *μ*g/L [19].

The intake of dietary iodine appears to be fairly well established in the young based on urinary excretion of iodide and iodide in breast milk and formula. The resulting serum levels of iodide in the young are less known because only in the last decade have advanced analytical methods been used to measure the inorganic iodide directly. Understanding the circulating levels of inorganic stable iodide is important for understanding the influence of dietary iodide on the radiation health risks (thyroid radioiodide dose) of infants and children exposed to radioiodine. The dose of radioiodide to the thyroid gland can be predicted mathematically based on competitive inhibition of radioiodide by stable iodide at the NIS protein of the thyroid gland.

### 2.5. Thyroidal Iodide and HPT Function

There are limited data on the total iodide content of thyroid glands in adults, children, and newborns (Table 3). Iodide content of thyroid glands provides an indicator of the status of the HPT axis. For euthyroid adults the thyroidal iodide stores (organified iodide) are estimated to be between 10 and 20 mg [25]. In Venezuelan population, Zabala et al. reported a median of 15 mg of thyroidal iodide content for adults, with a range of 4 to 37 mg of thyroidal iodide (Table 3) [20]. Fisher and Oddie reported a mean value of 15 mg iodide in thyroids of 8 young adults from the United States with a range of 9.0 to 23.6 [22]. In Russia, where mild iodide deficient conditions exist, lower thyroidal iodide values of 3.9 to 8.3 mg were found for adults and in teenagers, 1.5 mg [21] (Table 3). Thyroidal iodide stores have been measured in premature or term infants who only survived for a short period after birth (Table 3). A mean thyroidal iodide value of 0.29 mg for newborns is reported from Canada, where dietary iodide is sufficient [26]. Other studies in Europe reported thyroidal iodide concentrations of 0.09 and 0.04 mg for term babies [23, 24] (Table 3), where iodide insufficient conditions exist.

Thyroidal iodide stores are useful indicators of the status of the HPT axis, but data is difficult to obtain in humans. More age-specific information is needed on the thyroid gland, including thyroidal iodide content, weight, and the content of thyroid hormones and their precursors.

The HPT axis is very dynamic in the first hours and days after birth (Table 4). At birth serum thyroid stimulating hormone (TSH) and thyroxine (T4) concentrations are very high. This suggests that the negative feedback system is ineffective and that cold stimulation of the newborn may be the dominant factor controlling the HPT axis [58]. The calculated T4 secretion rate from the thyroid gland of the newborn is very high (Table 4). However, this condition is transient and a steady decline in serum TSH and T4 concentrations and T4 secretion occurs over several days after birth and the negative feedback becomes functional. Elevated secretion rates of T4 persist for months after birth.

Establishing population based reference intervals for serums TSH, T4, fT4, and T3 in newborns, infants, and children from the United States is needed along with clinical studies which can be used to quantitatively estimate thyroid secretion rates of thyroid hormones and deiodination rates (thyroid hormone metabolic rates) for euthyroid populations. This information would provide baseline data for better understanding the consequences of KI administration.

In conclusion, the infant and child homeostatic control of the HPT axis is very different than in the adult. Therefore, when considering the protection of infants and children from the harmful effects of thyroidal radiation, the age-dependent changes in physiology and the dynamic nature of the HPT axis need to be accounted for in estimating thyroidal radioiodine dosimetry.

### 2.6. KI Treatment (Table 5)

Safety concerns have been expressed regarding KI administration to infants and children while also ensuring adequate protection from radioiodine exposure [59]. The effectiveness of KI to reduce systemic radioiodide uptake into the thyroid gland in a clinical environment is inferred by evaluating the reduction in fractional RAIU. The use of KI in the young requires special consideration. The pharmacokinetic profiles for iodide are age-dependent, in part, because of physiological differences, but more importantly, the HPT axis differences between the infant and child and the adult are pronounced as discussed above.

Chronic treatment with iodine for goiter has caused hyperthyroidism [59]. Hypothyroidism has also occurred from chronic treatment with iodine and is associated with autoimmunity [59]. Spallek et al. reviewed the results of adverse effects from repeated doses with KI, iodide deficient population sensitivities to iodine, case studies of adverse effects, and the use of KI in Poland after the Chernobyl nuclear power plant accident [59]. They concluded that newborns and young children are most vulnerable to radioiodines and are also the group who experience a low frequency of adverse effects from KI. The adverse health risks from KI depend on nutritional iodine status. Iodine deficiency is a risk factor. However, the authors state that KI is an underrepresented research topic. Thus, the safe use of KI warrants more attention, particularly in infants.

The blocking effects of stable iodide salts (KI or sodium iodide (NaI)) on RAIU have been evaluated in adult human clinical studies, but clinical studies in children or infants are limited [60]. Ramsden et al. evaluated a few adult individuals with doses of 5 to 247 mg KI (Table 4) and reported greater than 86% RAIU inhibition at 30 hr for all doses except for the 5 mg KI (54% RAIU inhibition) [30]. At 3 or 5 days after dosing, the percent of RAIU inhibition was 16 to 22%. In another study [31] (Table 5) using several adult volunteers, 100 or 200 mg NaI was administered together with tracer radioiodine resulting in average 24 hr RAIU inhibition values ranging from 98 to 99% for both NaI doses. The RAIU inhibition drops to 50% if NaI was administered 3 hr after exposure to radioiodine. The effectiveness of NaI drops substantially after radioiodine is administered because the thyroidal radioiodide is organified (iodination of thyroglobulin) and stored in the thyroid gland. The iodinated thyroglobulin is transformed into thyroid hormones and slowly secreted from the thyroid gland in adults. The duration of effectiveness of a single dose is less than a day because of the renal clearance of iodide; thus, the authors recommend daily repeated doses of 100 to 200 mg NaI for radiation exposure situations exceeding one day. On a mg/kg body weight basis, this equates to 1.43 to 2.86 mg/kg KI administration for a 70 kg individual. These authors dosed a small number of individuals with other doses of KI (5, 25, 50, and 1000 mg) at the same time as administration of radioiodine. Although the study was not designed for dose-response assessment of KI, all doses were effective except for possibly the 5 mg of KI (RAIU inhibition value of 78%) (Table 5).

Sternthal et al. reported 24 hr RAIU inhibition values of over 92% for KI doses of 30, 50, and 100 mg and 36% RAIU inhibition for 10 mg KI (Table 4) [16]. In a more recent study, Hänscheid et al. reported 24 hr RAIU inhibition values of 64% for 7 individuals administered 100 mg KI 2 hr after administration of radioiodine [17]. The effectiveness of KI plummeted as the time interval was increased between administration of radioiodine and KI. Administration of KI before radioiodine administration was very effective, even 24 hr before administration of radioiodine. Takamura et al. reported 24 hr RAIU inhibition values of 73 and 80% in 8 hyperthyroid individuals dosed with 50 and 100 mg of KI, respectively [32].

Data on the ability of KI to block thyroidal uptake of radioiodide is considerable in adults, including varying conditions for time intervals between exposure to radioiodines and administration of KI and repeated dosing with KI. KI is very effective at reducing the thyroid gland burden of radioiodines if the treatment is administered within hours as of the exposure to radioiodines. If radioiodine exposure is prolonged repeated treatment with KI may be advantageous. Dose adjustment of KI to account for body weight is probably not needed in healthy nonpregnant, nonlactating adults if kidney function and nutrition are normal (e.g., 130 mg KI [45]).

For pediatric populations, the minimum effective KI dosages required for newborns, infants, and children are less certain because of a lack of adequate clinical studies. A clinical children's study was conducted in the 1960s, during the cold war at a state hospital in the United States, to determine the minimal effective dose of NaI to protect their thyroid glands from radioactive iodide irradiation [12]. Unfortunately the details of the study and study results are sparsely reported. The dose-responses for NaI and RAIU values were reported as *μ*g stable iodide per M^2^ skin surface area per day. For five 2-year-old children, baseline 24 hr RAIU values were collected and then over 8 weeks of daily administration of 0.3 mg stable iodide four 24 hr RAIU values were collected before the stable iodide dose was increased to 0.6 mg for a 4-week period in the same children and two 24 hr RAIU values were collected. NaI administration was curtailed and a final RAIU value was determined 2 weeks after NaI administration. RAIU inhibition values ranged from approximately 20 to 55% during 8 weeks of daily doses of 0.3 mg stable iodide and near 47 to 67% during 4 weeks of daily administration of 0.6 mg stable iodide. To convert dosing units from mg/M^2^ to body weight (mg/kg) Center for Disease Control and Prevention (CDC) body weight growth charts for children from the United States [61] were used to represent the possible range of body weights. A 5th percentile for a 2-year-old female child (lowest body weight) and a 95th percentile for a 2-year-old male child (largest body weight) were selected to represent the range of body weights of 2-year-old children reported in [12]. Surface area for different age groups per sex was taken from [62]. Three-tenths of one mg stable iodide/child (0.3 mg) equates to a stable iodide dose range of 0.03 to 0.02 mg/kg/day for a 2 yr infant and for 0.6 mg, 0.04 to 0.06 mg/kg/day.

Cuddihy reported that a KI dose of 1.8 mg repeated daily doses for 14 days in two children, 8 and 9 years old, resulted in 24 hr RAIU inhibitions of 33 and 48% [15], which would be for an estimated dose range of 0.05 to 0.06 mg/kg/day (using CDC growth charts). Noteboom et al. performed KI studies in infant chimpanzees as a surrogate for children. Six infant chimpanzees, aged two weeks to 2 years, had baseline RAIU values of 3.1 to 32.8%, with a mean value of 11.5% [63]. Mean RAIU inhibition at 24 hr for doses of 0.5 (*n* = 4), 1.5 (*n* = 3), and 5.0 mg/kg (*n* = 6) of KI was 74, 95, and 93%, respectively. Body weights were not given for the infant chimpanzees; however assuming a weight range of 2 to 4 kg [64], this equates to doses of 1-2, 3–6, and 10–20 mg of KI, respectively, which resulted in 93 to 95% RAIU inhibition for the 1.5 and 5.0 mg/kg dose groups and 74% for the 0.5 mg/kg dose group.

Single KI doses were administered to 10.5 million children in Poland in response to the Chernobyl nuclear disaster. Newborns received 15 mg iodine, children under 5 years of age were given 50 mg iodine, and all other children were administered 70 mg iodine [65]. KI administration occurred days after the onset of exposure to radioactive iodines. In children of 5 years and younger, RAIU inhibition of inhaled and ingested radioactive iodide-131 (^131^I) was estimated to range from 40 to 14%. The dose of KI for a newborn was approximately 6.7 to 3.5 mg/kg using a 5th percentile body weight for girls and 95th percentile body weights for boys in the United States. Less than 1% of newborns exhibited hypothyroidism (12 of 3214 babies) or increased serum TSH levels and decreased serum total thyroxine levels [65]. The predominant side effects for children were gastrointestinal (vomiting, stomach ache, and diarrhea), skin rashes, headache, and shortness of breath. Children under one year of age had the highest committed doses of thyroidal ^131^I^−^. An increased sensitivity of young children to thyroid cancer was observed [42, 43, 66], although the influence of iodine deficiency may be a contributing factor for thyroid cancer rates [67]. The proceedings from a workshop on iodine nutrition and radioactive iodide after Chernobyl incident state that, for a variety of reasons, it is difficult to determine the influence of iodide nutrition on the risks of thyroid cancer from exposure to radioactive iodine [44].

To better understand the effectiveness and safety of KI administration to infants and children more research is required, perhaps clinical studies. Alternatively, mathematical models have been developed to better understand radioiodine dosimetry in children. Compartmental mathematical models for predicting the dosimetry of radioiodide have existed for decades, primarily for adults [68]. More recently Zanzonico created a compartmental radioiodide model for children [69]. This nonphysiological model uses first order terms to account for age-dependent thyroidal uptake of radioiodide and secretion of organified radioiodide (thyroid hormone) from the thyroid gland. The model predicted that the mean ^131^I^−^ absorbed thyroidal dose in newborns is 26 times greater than adults. In another modeling study Jang et al. created a compartmental model for KI and radioiodide in children over 3 months of age [70]. These authors used first order terms to describe thyroidal uptake of stable iodide and radioiodide without considering age-dependent kinetic behavior. A first order term, describing secretion of organified radioiodide (thyroid hormone), was assumed to be age-dependent. The model predicted that KI would be equally effective at blocking thyroidal uptake of radioactive iodide for ages of 3 months to adulthood. Future modeling efforts, using physiological models [71] for the infant and child, and known information about the age-dependent physiology and the HPT axis (Tables 1 to 5) should provide useful insights into the effectiveness of KI as a drug to treat exposure to radioiodines.

### 2.7. What Do We Know about KI Dosing in Infants and Children?

To protect people from radioiodines the US Food and Drug Administration [45] recommends a single oral dose of 16 mg KI for neonates from birth to 1 month of age (7.1 to 3.7 mg/kg KI for 5th percentile female body weight and 95% percentile male body weight), 32 mg KI (7.0 to 3.6 mg/kg KI) for infants 1 month of age to children 3 years of age, and 65 mg KI for children from 3 years of age to up to 18 years old (5.7 to 0.6 mg/kg KI). In the case of adults, 130 mg KI per day is recommended, averaging about 2 mg/kg. The recommended KI dosages for the infant and child, on a mg/kg basis, would typically be greater than for an adult.

The two repeated dosing KI studies in children with small doses of KI [12, 15] are not adequate to draw general conclusions about the safety and efficacy of single high dose KI administration in children. The studies do suggest that small doses of KI result in RAIU inhibition. Since infants and children have a hyperactive HPT axis (Tables 2, 3, and 4), the evaluation of the efficacy of KI in hyperthyroid adults is useful for speculating on the efficacy of KI in children. The baseline RAIU in hyperthyroid, iodide sufficient adults from Japan was 65% of the administered radioiodide dose, while in the United States euthyroid (iodide sufficient) adult baseline RAIU values ranged from 17 to 50% [9, 16, 31]. For hyperthyroid iodide sufficient children from the United States Fields et al. reported RAIU baseline values of 72 to 99%, while several authors report a wide range of baseline RAIU values (40 to 70%) for iodide sufficient euthyroid children (Table 2) [9]. Twenty-four hr RAIU inhibition values of 73 and 80% for KI doses of 50 and 100 mg were reported for iodide sufficient adult hyperthyroid Japanese subjects [32] and 94 to 99% for KI doses of 50 and 100 mg [16, 31] in iodide sufficient euthyroid adult volunteers from the United States.

These results demonstrate that KI is less effective at blocking thyroidal uptake of radioiodide if the HPT axis is hyperactive in adults. By analogy KI is expected to be less effective in infants and children with a “hyperactive HPT axis.” The Poland experience, where very large numbers of children were administered doses of 15 to 50 mg KI, resulted in apparently low RAIU inhibition for children less than 5 years of age [65]. Interpreting this study is difficult because of confounding factors. These children may have been iodide deficient, which would further exacerbate the hyperactive HPT axis and further reduce the effectiveness of KI. Also the time lag from the onset of radioiodine exposure to KI treatment was probably detrimental, further reducing the effectiveness of KI. Interestingly the authors of the infant monkey KI study [63] suggest that a dose of at least 1.5 mg/kg (5.1 mg KI for a newborn human) is needed to protect the infant thyroid gland.

## 3. Recommendations

The US FDA recommended single KI doses for infants may effectively block radioiodides [45]. Clearly, more research is needed to better characterize the safety and effectiveness of KI in infants and children. Several issues need to be considered when addressing administration of KI in the young. A low incidence of thyroid and nonthyroid related side effects may occur. The protective effect of KI will be for a shorter duration than in adults because of the hyperactive nature of the HPT axis. Stable iodide will be excreted into urine more quickly reducing the protective effect of KI. The amount of thyroidal iodide stores is much less in infants than adults. The half-life of organified iodide in the thyroid gland of children (~2.5 or 3 days) is less than the radionuclide decay half-life of ^131^I (8 days), which is not the case in adults. Thus, radioactive thyroid hormones would be secreted from the thyroid gland and distributed into the body. Radioiodide derived from deiodination of radioactive thyroid hormones would be secreted in urine or recycled back into the thyroid gland, increasing the duration of systemic radioiodide. As the lag time from radioiodine exposure to administration of KI increases, the effectiveness of KI would be expected to decrease at a faster rate than in adults because of the hyperactive nature of the HPT axis, raising an important question about “single versus multiple doses of KI.” The iodide nutritional status of the sensitive population is expected to alter the effectiveness of KI.

To conduct KI clinical trials in children seems unattainable; however, the Presidential Commission of Bioethical Issues did recently provide research guidelines to the Secretary of Health and Human Services for considering medical countermeasure pediatric trails for an anthrax vaccine [72]. Preevent pediatric testing of medical countermeasures may be carried out by clinical trials using age deescalation, coupled with previous informative research endeavors such as mathematical modeling, toxicity testing of laboratory animals, and adult human studies.

With over 100 operational nuclear reactors in the USA, continued planning and reevaluation for the possible use for KI is prudent. Advanced research methods for toxicity testing and computational modeling of the thyroid system [71, 73–76] and extrapolation tools [77] can provide useful quantitative predictions of pediatric KI doses which will maximize the blocking effect of KI while protecting against adverse outcomes from KI. These research findings can then be compared to existing recommended pediatric KI doses to ensure the safety of children.

## Figures and Tables

**Table 1:** Selected physiology values in infants, children, and young adults.

	Age (days)	Value	Age (months)	Value	Age (years)	Value	Age (years)	Value	References
Blood flows (L/hr/kg bw)^a^									
Cardiac output	6	11.1^b^	10.6	8.6^c^	6	11.1	18	8	[1, 2]
Alveolar ventilation	1	13.4	3	8.7	6	6.9	18	6.7^d^	[3]
Glomerular filtration	2	0.09^b^	12	0.18	3	0.17	12	0.13	[4]
Blood flow to brain	1	3.2	12	4.3	5	2.9	15	0.83	[5]
Blood flow to kidney	1	1.9	12	1.4	5	1.8	15	1.1	[5]
Blood flow to thyroid	—	—	—	—	—	—	18	0.13^e^	[6]
Organ volumes (L/kg bw)^a^									
Body weight (Kg)	1	3.4	12	9.8	6	21.1	18	54.4	[7]
Blood	1	0.088	24	0.046	6	0.081	18	0.077	[7]
Brain	1	0.10	12	0.091	6	0.057	18	0.024	[7]
Kidney	1	0.007	12	0.0066	6	0.0052	18	0.0044	[7]
Thyroid	1–30	0.3–0.4*E* − 3	12–24	0.2–0.3*E* − 3	5–9	0.2–0.3*E* − 3	15–19	0.3*E* − 3	[8]

^a^Body weight for females at specified ages calculated using equation from [7].

^b^Calculated using body weight at birth.

^c^Calculated using body weight of 1 year old.

^d^Derived values using respiratory frequency, tidal volume, and physiological dead space [3].

^e^Calculated based on reported percentage of blood flow to thyroid in adult humans (1.6% of cardiac output) [6] and cardiac output (8 L/hr/kg bw) in females of 18 years old.

**Table 2:** Radioactive iodide uptake into the thyroid gland (RAIU, expressed as percent of the administered radioactive dose) and thyroidal and renal clearance rates of iodide in infants, children, and adults.

RAIU study	24 hr RAIU % of dose mean	24 hr RAIU % of dose range	Number of volunteers	Location	Reference
Infant and child age					
2 months to 18 years	31.1	17 to 50	60	United States	[9]
9 to 18 years, hyperthyroidism	87	72 to 99	5	United States	[9]
Newborn, 2-3 days, 14^a^ hr RAIU	69.7^a^	46 to 97^a^	7	United States	[10]
Premature (2 lb, 4 oz to 5 lb 15 oz)	38.6	28 to 52	16	United States	[11]
2 years	—	15–23	5	United States	[12]
9 to 15 years	52.9	28–52	26	Belgium	[13]
1.5 to 2 years (euthyroid)	39.6	SD ± 3.08	6	Republic of Senegal	[14]
1.5 to 2 years (malnutrition)	23.9	SD ± 6.24	12	Republic of Senegal	[14]
7 to 9 years	23.5	21 to 25	4	United States	[15]
Adults					
Euthyroid	32.2	16 to 50	64	United States	[9]
Hyperthyroid	76.1	34 to 100	62	United States	[9]
23 to 50 years	20.0	17.2 to 21.8	22	United States	[16]
Unknown	41.7	SE ± 1.5	unknown	Belgium	[13]
25 to 46 years	24.8	10–47.8	27	Germany	[17]
18 to 57 years	20.0	SE 1.5 to 3.8	37	United States	[18]

	Mean (mL/min)	Mean (mL/min/kg)	Number of Volunteers	Location	Reference

Thyroidal clearance of iodide					
Children					
Birth to 3 weeks	—	2.52	3	Belgium	[19]
3 weeks to 6 months	—	1.09	4	Belgium	[19]
6 to 12 months	—	0.9	7	Belgium	[19]
1 to 2 years	—	1.7	7	Belgium	[19]
9 to 15 years, normal	30.3	0.80	26	Belgium	[13]
Normal	21.5	—	6	Republic of Senegal	[14]
Malnourished	7.3	—	12	Republic of Senegal	[14]
Adults	25.3	0.35	unknown	Belgium	[13]
Renal clearance of iodide					
Children					
Birth to 3 weeks	—	0.74	3	Belgium	[19]
3 weeks to 6 months	—	1.1	4	Belgium	[19]
6 to 12 months	—	0.73	7	Belgium	[19]
1 to 2 years	—	1.5	7	Belgium	[19]
9 to 15 years	25.5	0.65	26	Belgium	[13]
Adults	29.7	0.41	—	Belgium	[13]

^a^Refers to a 14 hr RAIU value, which is different than all others, which are in the column with a header stating 24 hr RAIU.

**Table 3:** Thyroid gland weights and thyroidal iodide stores in infants, children, and adults.

Subject	Thyroid weight (g)	Thyroidal iodide (mg/g)	Thyroidal iodide (mg)	Location	Reference
Adult males (*n* = 50)	10 ± 2 SD (5.6 to 17.7)	1.4 ± 0.7 median, SD (0.42 to 3.43)	15 ± 8 (4 to 37)	Venezuela	[20]
Age 16 to 85 years, range of mean values (*n* = 83)	13 to 15.3	0.32 to 0.67	3.9 to 8.3	Russia	[21]
Age less than 16 years, 8 years average age (*n* = 5)	6.1 ± 1.0 SE	0.24 ± 0.08 SE	1.5 ± 0.52 SE	Russia	[21]
Young adults (*n* = 8)	14.5 ± 1.2 SE (11.1 to 20.3)	0.97	15.0 ± 1.5 SE (9.0 to 23.6)	United States	[22]

Newborns					
Less than 13-day-old term newborns (*n* = 4)	0.74 ± 0.74 SD	0.060 ± 42.3 SD	0.04	Italy	[23]
Gestation age 22 to 34 weeks preterm (*n* = 28)	0.61 ± 0.35 SD	0.042 ± 0.04 SD	0.03	Italy	[23]
Less than 30-day-old near-term newborns (*n* = 15)	0.93 ± 0.4 SD	0.096 ± 0.034 SD	0.09	Yugoslavia	[24]
Preterm newborns, under 10 days of age (*n* = 9)	—	0.092 ± 0.01 SE 0.049 to 0.166	—	Belgium	[25]
Preterm newborns, under 10 days of age (*n* = 8)	—	0.270 ± 0.05 SE 0.094 to 0.493	—	Canada	[25]
Newborns (*n* = 13)	1.0	—	0.292 ± 0.05 SE	Canada	[26]
Preterm newborns (*n* = 17), under 20 hrs of age*, 1–3 days of age**, and 10 days to 7 weeks of age	0.42 to 1.2	0.06 ± 0.05* 0.09 ± 0.05** 0.26 ± 0.05	—	France	[27]

The use of ** and * is associated with ages of newborns shown to the left, in column 1.

**Table 4:** Estimated secretion rates of thyroxine (T4) and total and free serum concentrations of T4 in infants, children, and adults.

Subject	T4 secretion *μ*g/kg/day	Serum TSH mIU/L	Serum total T4 (nmol/L)/ Serum free T4 (pmol/L)	Reference
Adults	1.5	0.4 to 4.2	55–161/12–32	[28]
	0.75	—	—	[29]
Infant age				
1 to 4 days	10	1 to 39	142–277/28–68	[28]
7 to 28 days	7	1.7 to 9.1	106–221/12–30	[28]
1 to 12 months	6	0.8 to 8.2	76–210/10–23	[28]
Child age				
1 to 15 years	3 to 5	0.7 to 5.7	54–193/10–26	[28]
3 to 9 years	1.29	—	—	[29]

**Table 5:** Inhibition of radioactive iodide uptake (RAIU) into the thyroid gland by potassium or sodium iodide in children and adults.

Subject, KI dose (mg)	RAIU (% thyroidal radiotracer dose)	RAIU inhibition (% of radiotracer dose)	Reference location
Adult	*30 hr RAIU *	*RAIU % inhibition *	[30] England
247 (*n* = 1)	38	100 at 30 hr, 16 at 5 days	
124 (*n* = 1)	45	96 at 30 hr, 22 at 5 days	
37 (*n* = 1)	44	86 at 30 hr, 16 at 3 days	
25 (*n* = 1)	39	97 at 30 hr	
5 (*n* = 1)	52	54 at 30 hr	

Adult	*24 hr RAIU *	*24 hr RAIU % inhibition *	[16] United States
10 (*n* = 5)	19.4	36	
30 (*n* = 15)	19.4 to 22.6	93 to 96	
50 (*n* = 5)	19.7	92	
100 (*n* = 5)	17.2	96	

Adult, NaI	*24 hr RAIU *	*24 hr RAIU inhibition *	[31] United States
5 (*n* = 1)	33	78	
25 (*n* = 1)	41	96	
50 (*n* = 1)	28	97	
100 (*n* = 13)	28.6	97.8	
200 (*n* = 10)	25.9	98.5	
1000 (*n* = 1)	19	99.0	

Adult (hyperthyroidism)	*24 hr RAIU *	*24 hr RAIU inhibition *	[32] Japan
50 (*n* = 8 for both doses) 100	65.3 for 8 patients	73.3 79.5	

Children	*1-2 day RAIU *	*24 hr RAIU inhibition *	[15] United States
1.8 (*n* = 2, 8, and 9 years of age)	21 and 25	33 and 48	

Children (*n* = 5, 2 years of age)	*24 hr RAIU *	*24 hr RAIU % inhibition *	[12] United States
0.3	*≈*15 to 25	*≈*20 to 55	
0.6	*≈*15 to 25	*≈*47 to 67	
